# In Vitro Cytochrome P450 Interaction Profile and ADME Characterisation of Gold(I)–Triphenylphosphine Complexes with 6-Alkoxy-9-deazapurine Ligands

**DOI:** 10.3390/pharmaceutics18050599

**Published:** 2026-05-14

**Authors:** Martina Medvedíková, Ján Vančo, Zdeněk Trávníček, Pavel Anzenbacher

**Affiliations:** 1 Department of Pharmacology, Faculty of Medicine and Dentistry, Palacký University in Olomouc, Hněvotínská 3, CZ-779 00 Olomouc, Czech Republic; martina.medvedikova@upol.cz (M.M.);; 2 Institute of Molecular and Translation Medicine, Faculty of Medicine and Dentistry, Palacký University in Olomouc, Hněvotínská 5, CZ-779 00 Olomouc, Czech Republic; 3 Regional Centre of Advanced Technologies and Materials (RCPTM), Czech Advanced Technology and Research Institute (CATRIN), Palacký University in Olomouc, Šlechtitelů 27, CZ-779 00 Olomouc, Czech Republic; jan.vanco@upol.cz

**Keywords:** gold(I) complexes, 9-deazahypoxanthine, 9-deazapurine, cytochrome P450, ADME, isothermal titration calorimetry

## Abstract

**Background/Objectives**: Gold(I) complexes are promising bioactive agents with anticancer and anti-inflammatory potential. This study evaluated cytochrome P450 (CYP) interactions and in vitro pharmacokinetic properties of two Au(I)–triphenylphosphine complexes bearing 6-alkoxy-9-deazapurine ligands. **Methods**: Complexes [Au(HL_1,2_)(PPh_3_)] (HL_1_ = 6-isopropyloxy-9-deazapurine, complex **1**; HL_2_ = 6-benzyloxy-9-deazapurine, complex **2**) were investigated. Inhibition of nine human CYP isoforms was assessed in liver microsomes, and kinetics were analyzed using Dixon and Lineweaver–Burk plots. CYP binding was evaluated by UV–Vis difference spectroscopy. ADME properties (chemical/plasma stability, microsomal stability, plasma protein binding, and PAMPA permeability) were determined. Binding thermodynamics were analyzed by ITC. **Results**: Both complexes weakly inhibited most CYP isoforms, with stronger effects on CYP2C9 and CYP3A4/5. A non-competitive inhibition mechanism was observed, which may be related to the binding of the complexes to the substrate channels of CYP2C9 and CYP3A4, thereby limiting the active site’s accessibility to the substrate, as supported by molecular docking studies. UV–Vis spectra showed type I binding with *Kd* values of 9.32 µM (**1**) and 12.64 µM (**2**). Both compounds showed high chemical and plasma stability (>90%), moderate microsomal stability (~60% after 60 min), high plasma protein binding (~80%), and low passive permeability. **Conclusions**: Au(I)–triphenylphosphine complexes with 6-alkoxy-9-deazapurine ligands exhibit moderate CYP affinity and defined pharmacokinetic profiles, supporting further preclinical evaluation.

## 1. Introduction

Metal-based drugs are a key part of modern anticancer chemotherapy, with cisplatin setting the clinical benchmark despite limitations due to acquired resistance, cumulative toxicity, and DNA-centred mechanisms [1]. These limitations have driven the search for alternative metallodrugs that act through non-genomic pathways and offer improved safety profiles. Among non-platinum metal centres, gold complexes are particularly attractive because of their high affinity for soft biological nucleophiles, especially sulfanyl and selanyl groups in proteins [2].

**Figure 1:**
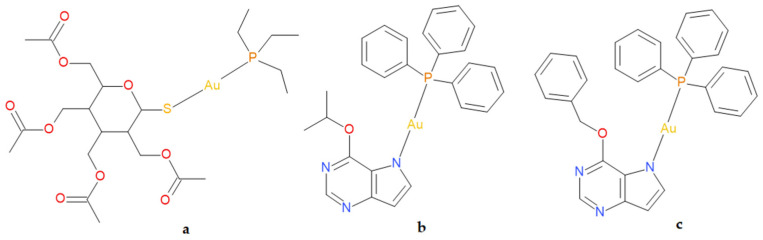
Structural formulas of Auranofin (**a**) and studied gold(I) complexes—complex **1** (**b**) and complex **2** (**c**).

The therapeutic potential of gold compounds was first clinically validated with Auranofin (2,3,4,6-tetra-*O*-acetyl-1-thio-*β*-*D*-glucopyranosato-*S*-(triethyl-phosphine)gold) [3] (Figure 1a), an orally administered gold(I) phosphine complex approved for rheumatoid arthritis [4]. Auranofin has recently attracted attention as an anticancer agent because it inhibits thioredoxin reductase (TrxR), a selenoenzyme involved in intracellular redox balance [5,6]. TrxR inhibition disrupts the thioredoxin system, induces oxidative stress and mitochondrial dysfunction, and promotes cell death through mechanisms distinct from DNA-targeting chemotherapy [7,8]. Auranofin is currently being evaluated, as a single agent or in combination therapies, in Phase I/II clinical trials for several cancers, including chronic lymphocytic leukaemia, recurrent ovarian cancer, and glioblastoma combination protocols [9].

Recent reviews emphasise that the biological performance of Au(I) complexes depends on rational ligand design, especially nitrogen-donor heterocycles able to modulate electronic properties, redox behaviour, and intracellular distribution [10,11,12]. Beyond TrxR inhibition, modern Au(I) systems can interfere with mitochondrial respiration, induce reactive oxygen species, and activate ferroptosis, necroptosis, or paraptosis [11,13]. Mitochondria-targeting Au(I)-NHC complexes have induced ferroptosis in three-dimensional tumour spheroids [14], while sterically demanding phenanthroline-based Au(I)-NHC complexes triggered non-apoptotic death and reduced metastatic potential in apoptosis-resistant lung adenocarcinoma cells [15]. These findings show that ligand architecture influences not only cytotoxic potency, but also mechanistic profiles and potential therapeutic selectivity.

Among gold-based candidates, linear two-coordinate Au(I) complexes of the general type [Au(L)(PR_3_)] are one of the most extensively investigated structural classes [16]. Their predictable geometry, affinity for soft sulfur and selenium donors, and tunable ligand environment enable rational modulation of physicochemical and biological properties [17]. The phosphine ligand and ancillary heterocycle both influence lipophilicity, redox stability, cellular uptake, and biological activity [18], while triphenylphosphine (PPh_3_)-containing Au(I) complexes combine sufficient physiological stability with controlled reactivity toward biologically relevant nucleophiles [19].

Purine-derived ligands represent bioinspired scaffolds in medicinal inorganic chemistry [20]. Their similarity to endogenous nucleobases may improve biological compatibility while preserving nitrogen-donor coordination. In particular, 9-deazapurine derivatives show modified electronic properties compared with canonical purines, providing enhanced coordination flexibility and tunable donor strength [21]. Replacement of the N9 atom of the imidazole moiety by carbon also eliminates *N*-glycosidic bond formation, which may improve metabolic stability of the resulting metal complexes [21].

Substitution at the 6-alkoxy-9-deazapurine scaffold provides an additional approach for tuning physicochemical and biological properties. Alkoxy groups can markedly influence lipophilicity, solubility, membrane permeability, and intracellular accumulation [22,23]. More hydrophobic benzyloxy substituents may improve membrane transport and retention, whereas smaller isopropyloxy groups may offer a more balanced solubility–permeability profile [24]. Although such structure–property relationships are well recognised for heterocyclic scaffolds, systematic experimental validation in phosphine-coordinated Au(I) complexes remains limited, particularly for compounds differing only in the alkoxy substituent.

In this structural context, the present study focuses on two linear phosphine-coordinated gold(I) complexes incorporating substituted 9-deazapurine ligands: complex **1** [Au(6-isopropyloxy-9-deazapurine)(PPh_3_)] (Figure 1b), and complex **2** [Au(6-benzyloxy-9-deazapurine)(PPh_3_)] (Figure 1c). These compounds retain the characteristic linear Au(I)-PPh_3_ framework but differ in the 6-alkoxy substituent, enabling evaluation of how subtle ligand modifications affect physicochemical behaviour, biological activity, and metabolic interactions.

The biological relevance of related gold(I) complexes bearing 6-alkoxy-9-deazapurine-type ligands was demonstrated by Vančo et al. [25]. Phosphine-coordinated Au(I) complexes were active against several cancer cell lines, including A2780, A2780R, MCF-7, HOS, HeLa, A549, G361, 22Rv1, and THP-1, with lower toxicity toward non-malignant HEP202 cells. Their IC_50_ values were generally in the low micromolar range and were comparable or superior to cisplatin in selected models. In addition to antiproliferative effects, the complexes also reduced pro-inflammatory mediators in stimulated macrophages, supporting a dual antitumor and anti-inflammatory profile and providing a rationale for further modification of the 9-deazapurine scaffold.

Although gold compounds are widely investigated for cytotoxicity and redox-related mechanisms, their pharmacokinetic properties and metabolic liabilities remain less explored [26,27]. Pharmacokinetic behaviour determines systemic exposure, tissue distribution, clearance, and drug–drug interaction risk, yet comprehensive ADME profiling of Au(I) and Au(III) complexes is limited [28]. After systemic administration, gold complexes can form stable plasma-protein adducts involving albumin and transferrin [29], and Au(I) speciation may be altered by thiol-triggered ligand exchange with glutathione and related low-molecular-weight thiols in vivo. Recent analyses indicate that ligand architecture influences chemical and metabolic stability, plasma protein binding, and susceptibility to biotransformation [30], but CYP450 modulation by gold-based agents remains insufficiently characterised despite its relevance for systemic exposure and drug–drug interaction risk.

Cytochrome P450 (CYP450) enzymes are the main phase I metabolic system for xenobiotics and mediate oxidative biotransformation of a large proportion of clinically used drugs [31,32]. These heme-containing monooxygenases are expressed mainly in the liver, with important extrahepatic expression in the intestine, lung, kidney, and selected tumour tissues [33]. Clinically relevant isoforms such as CYP3A4/5, CYP2D6, CYP2C9, CYP2C19, and CYP1A2 influence systemic clearance, oral bioavailability, and interindividual pharmacokinetic variability [34,35]. Even moderate CYP3A4 inhibition can substantially alter drug exposure because of its dominant role in hepatic and intestinal metabolism [36].

Mechanistically, CYP450 inhibition can be competitive, non-competitive, or mechanism-based, each with distinct kinetic and clinical implications [37]. Metal-containing compounds are relevant in this context because they may interact with the heme iron, coordinate active-site residues, or induce conformational changes [38,39]. Recent reviews indicate that inorganic systems, metal complexes, and nanoparticles can also alter CYP450 expression or reductase interactions; for example, iridium, ruthenium, and copper complexes inhibit selected CYP isoforms through different mechanisms [40]. Owing to their affinity for sulfanyl and selanyl groups, Au(I) complexes may interact with cysteine residues near catalytic domains [41,42], and the lipophilicity of phosphine-containing Au(I) complexes may favour accumulation in the endoplasmic reticulum, where microsomal CYP450 enzymes are located [43].

This study was motivated by findings that the title complexes [Au(L_1,2_)(PPh_3_)] (**1** and **2**) demonstrated notable in vitro anticancer activity and selectivity, as well as anti-inflammatory activity comparable to Auranofin and reduced L-carrageenan-induced oedema in rats in vivo [25]. Based on these results, the compounds represent feasible candidates for further antitumor and anti-inflammatory evaluation. We therefore aimed to clarify structure–property relationships within the 6-alkoxy-9-deazapurine scaffold and assess their influence on CYP450 activity and selected ADME-related parameters. In contrast to previous studies on Au(I) complexes, which have largely focused on biological activity without detailed metabolic characterization, this study integrates CYP450 interaction profiling with comprehensive in vitro pharmacokinetic evaluation. Importantly, it seeks to clarify whether CYP450 modulation is driven by the intact metal complex or by its individual components, and to establish a mechanistic framework for these interactions using complementary biophysical and enzymatic approaches. By combining these perspectives, the study provides new insight into the role of metal coordination in governing CYP450 interactions and pharmacokinetic behavior, thereby contributing to a more rational design of gold-based metallodrugs and a better assessment of their translational potential and safety profile.

## 2. Materials and Methods

The synthesis and thorough characterization of gold(I)-triphenylphosphine complexes, [Au(HL)(PPh_3_)], where HL_1_ = 6-isopropyloxy-9-deazapurine (complex **1**) (Figure 1b) or HL_2_ = 6-benzyloxy-9-deazapurine (complex **2**) (Figure 1c), including the analytical data regarding the purity of the compounds, were described previously in the literature [25]. The gold(I) complexes used in this work were obtained from the same batch reported in the literature. Thus, the final formulas of the studied complexes were already confirmed and well known based on thorough characterisation of the compounds.

Cryopreserved pooled human liver microsomes were obtained from Xenotech (Lenexa, KS, USA), and human plasma was obtained from the Transfusion Department of University Hospital Olomouc (Olomouc, Czech Republic).

For determination of CYP450 activities, 7-ethoxyresorufin and 7-ethoxy-4-(trifluoromethyl)coumarin were purchased from Fluka (Buchs, Switzerland). Coumarin, testosterone, diclofenac, bufuralol, and chlorzoxazone were obtained from Sigma-Aldrich (Prague, Czech Republic), midazolam was purchased from Abcam (Cambridge, UK), paclitaxel was obtained from Chemos CZ (Prague, Czech Republic) and (*S*)-mephenytoin was purchased from SantaCruz Biotechnology Inc. (Heidelberg, Germany).

Dimethyl sulfoxide (DMSO) and potassium dihydrogen phosphate were obtained from Lach-Ner (Neratovice, Czech Republic); dichloromethane, methanol and acetonitrile were from VWR Prolabo (Fontenay-sous-Bois, France). All other chemicals were supplied by Sigma Aldrich CZ (Prague, Czech Republic).

### 2.1. In Vitro Pharmacological Properties

The pharmacological profiles of complexes **1** and **2** were evaluated under in vitro conditions, including assessment of chemical stability, plasma stability, microsomal stability, plasma protein binding, and permeability across an artificial membrane. All assays were performed according to previously published protocols [44,45,46].

Quantitative analysis of the samples was carried out using an Agilent RapidFire 300 High-Throughput Mass Spectrometry system (Agilent Technologies, Wakefield, MA, USA) coupled with a QTRAP 5500 mass spectrometer (AB Sciex, Concord, ON, Canada) (RF-MS platform). Samples were aspirated directly from microplates into a 10 µL sample loop and transferred onto C4 solid-phase extraction cartridges. The cartridges were washed with solvent A (a mixture of 5% acetonitrile and 95% water with 0.1% formic acid) at a flow rate of 1.5 mL/min for 3 s to remove salts and matrix components. Subsequently, the retained analytes were eluted with solvent B (a mixture of 95% acetonitrile and 5% water with 0.1% formic acid) at a flow rate of 0.4 mL/min for 5 s into the mass spectrometer.

Mass spectrometric analysis was carried out using electrospray ionization (ESI) in positive ion mode. Specific daughter ion transitions were monitored via a multiple reaction monitoring (MRM) approach for precise and sensitive quantification of the analytes.

### 2.2. Cytochrome P450 Activities

The enzyme activities of the individual CYP450 isoforms were determined according to established protocols [47]. Preliminary kinetic experiments to determine the Michaelis constant (*Km*) and the limiting velocity (*V*max) were performed. These experiments were used to establish incubation times within the linear range of product formation and to select substrate concentrations corresponding to the *Km* range. The amount of HLM (expressed as the amount of CYP450 in pmol and concentration of HLM protein in mg/mL in the reaction vessel) in the reaction (with dilutions based on the established protocols of CYP450 activities measurements) as was follow: The CYP1A2 assay was based on 7-ethoxyresorufin *O*-deethylation with 35 pmol of CYP450 and *Km* of 1.56 µM, the CYP2A6 assay on coumarin 7-hydroxylation with 35 pmol of CYP450 and *Km* of 14 µM, the CYP2B6 based on 7-ethoxy-4-(trifluoromethyl)coumarin *O*-deethylation with 35 pmol of CYP450 and *Km* of 15.25 µM, activity of the CYP2C8 enzyme based on the paclitaxel 6-hydroxylation with 70 pmol of CYP450 and *Km* of 18.41 µM, of the CYP2C9 was based on diclofenac 4’-hydroxylation with 35 pmol of CYP450 and *Km* of 16 µM, CYP2C19 assay was based on (*S*)-mephenytoin 4’-hydroxylation with 50 pmol of CYP450 and *Km* of 28 µM, the CYP2D6 based on bufuralol 1’-hydroxylation with 70 pmol of CYP450 and *Km* of 14.30 µM, CYP2E1 activity was assessed by chlorzoxazone 6-hydroxylation with 160 pmol of CYP450 and *Km* of 56 µM; CYP3A4 activity was determined based on the testosterone 6β-hydroxylation with 10 pmol of CYP450 and *Km* of 100 µM and midazolam by 1’-hydroxylation with 13 pmol of CYP450 and *Km* of 2.2 µM.

Incubation mixtures were prepared in 0.1 M potassium phosphate buffer (pH 7.4) and supplemented with an NADPH-generating system composed of isocitrate dehydrogenase (6 U/mL), NADP^+^ (0.5 mM), isocitric acid (4 mM), and MgSO_4_ (5 mM). Tested complexes, free ligands, and triphenylphosphine were prepared from 10 mM stock solutions in dimethyl sulfoxide (DMSO) and diluted to the required final concentrations. Because DMSO may interfere with CYP450 activity, solvent controls containing DMSO without inhibitors were included in all experiments, and the final DMSO concentration was maintained below 0.1% (*v*/*v*). Inhibition studies were conducted using five concentrations of the tested compounds, ranging from 10 to 100 µM. The upper concentration limit was selected to enable reliable determination of inhibitory potency and, when applicable, calculation of IC_50_ values. Incubations were performed at 37 °C in two independent experimental series, with each condition analysed in triplicate. The preincubation of the reaction mixture with potential CYP450 inhibitors was performed according to established protocols at 37 °C [48]. Monitoring of the metabolites formed from specific substrates was done by HPLC using the Prominence system (Shimadzu, Kyoto, Japan) equipped with a LiChroCART 250-4 LiChrospher 100 RP-18 column or Chromolith^®^ HighResolution RP-18 endcapped column (Merck, Darmstadt, Germany) or Kinetex Biphenyl column (Phenomenex, Torrance, CA, USA). Inhibitory effects of the studied complexes, free ligands, and triphenylphosphine on individual CYP450 activities were evaluated by plotting residual enzyme activity as a function of inhibitor concentration. The IC_50_ values were calculated from nonlinear regression analysis of residual activity versus the logarithm of inhibitor concentration using GraphPad Prism 6 (GraphPad Software, Boston, MA, USA).

The apparent *Ki* values were obtained from additional measurements at substrate concentrations of 1/2 *Km*, *Km*, 2 *Km*, and 4 *Km* for inhibition. Inhibition of individual CYP450 activities was assessed by plotting the remaining activity against the inhibitor concentration using GraphPad Prism 6 (GraphPad Software, Boston, MA, USA). Throughout the paper, the apparent *Ki* values are reported. The inhibition data were fitted to various enzyme inhibition models using nonlinear regression in GraphPad Prism. The appropriate model for each data set was selected based on visual inspection of the Lineweaver-Burk and Dixon plots. In addition to the inhibition studies described above, a series of single-point assays was performed to detect potential slow-binding or irreversible inhibition, which may arise from oxidative modification of microsomal proteins. For this purpose, two parallel experimental setups were employed. In the first set of samples, human liver microsomes (HLMs), used at a concentration ten times higher than that applied in standard activity experiments, were incubated with the tested complex (final concentration of 25 µM) or with 1% DMSO as a solvent control. The samples were preincubated at 37 °C for 30 min in the presence of an NADPH-generating system. In the second set of samples, HLMs were preincubated with the tested complexes or 1% DMSO at 37 °C for 30 min in the absence of the NADPH-generating system. The NADPH-generating system was added only after the preincubation step. Following preincubation, an aliquot of each sample was withdrawn, diluted tenfold with the reaction buffer, and the corresponding substrate was added at a concentration of 5 *Km*. The residual microsomal activity was then determined as described above and compared with the activity of uninhibited microsomes. Time-dependent inhibition was inferred when a decrease in enzymatic activity was observed exclusively in samples preincubated with the NADPH-generating system. To exclude potential solvent-related effects, appropriate solvent control samples were analysed.

### 2.3. Spectroscopic Study of Interactions of the Studied Complexes **1** and **2** with Human Liver Microsomes

Difference spectral measurements were performed to evaluate interactions between the studied complexes and human liver microsomes (HLM) according to the method described by Schenkman J. and Jansson I. [49]. Microsomal suspensions were prepared in 50 mM potassium phosphate buffer (pH 7.4) and adjusted to a final cytochrome P450 concentration of 1 µM in the cuvette. The tested compounds were dissolved in the same buffer, with their final concentrations in the assay ranging from 0.002 to 33.31 µM. Spectral changes were observed at room temperature using a Varian Cary 500 UV–Vis spectrophotometer (Varian, Palo Alto, CA, USA). Repeated scans were recorded within the wavelength range of 300–700 nm.

Difference spectra were evaluated by monitoring absorbance changes within the Soret region (370–450 nm). The magnitude of spectral shifts was plotted against the compound concentration. Data were processed and analysed using GraphPad Prism 6 (GraphPad Software, La Jolla, CA, USA).

### 2.4. Isothermal Titration Calorimetry (ITC)

The interactions of the studied gold(I) complexes with selected cytochrome P450 isoforms were investigated by isothermal titration calorimetry (ITC). Specifically, human bactosomes of CYP1A2, CYP2A6, and CYP3A4, as well as recombinant human CYP3A4, were employed as protein targets.

All ITC experiments were performed at 25 °C using a Nano ITC Low Volume calorimeter (TA Instruments, New Castle, DE, USA). “The compound solution under study, at a total concentration of 16 µM in the syringe, was injected in 20 consecutive injections of 2.5 µL aliquots into the calorimetric cell containing 250 µL of the respective protein solution at a concentration of 2 µM.” The time interval between consecutive injections was set to 300 s, and the stirring speed was maintained at 250 rpm throughout all measurements.

All solutions were prepared in 100 mM phosphate buffer (pH 7.4) supplemented with 1% (*v*/*v*) dimethyl sulfoxide (DMSO) to ensure adequate solubility of the compounds and were thoroughly degassed prior to use. Control experiments were performed by titrating each compound solution into the buffer alone under otherwise identical conditions. The heat contributions obtained from these control titrations were subtracted from the corresponding experimental data to yield corrected thermograms.

The corrected thermograms were analysed using the independent binding model implemented in the NanoAnalyze software (TA Instruments, New Castle, DE, USA) to obtain the thermodynamic parameters of the interaction, including the binding constant (*K*a), binding enthalpy (Δ*H*), entropy contribution (Δ*S*), and stoichiometry (*n*).

### 2.5. Geometry Optimisation of Complex ***2***

While the molecular structure of complex **1** has been previously reported [25], no X-ray crystallographic data for complex **2** are available in the literature. Therefore, the geometry of complex **2** was optimised using density functional theory (DFT). All calculations were performed with the Spartan’20 software package (Wavefunction Inc.: Irvine, CA, USA). The geometry optimisation was carried out using the B3LYP hybrid density functional and LANL2DZ basis set for all atoms, including the corresponding effective core potential for the heavy Au atom. The purpose of these calculations was primarily to obtain a reliable representation of the molecular geometry of complex **2**, rather than to provide a detailed theoretical description of its electronic structure.

### 2.6. Molecular Docking Procedure

Molecular docking was used to test the hypothesis that complexes **1** and **2** can act as non-competitive inhibitors by blocking the substrate’s access to the active sites of CYP450. As targets, the X-ray diffraction-based protein structures of CYP2C9 and CYP3A4 were used. The CYP2C9 structure for the docking procedure was prepared from the chain A of the inclusion complex of CYP2C9 with a specific inhibitor (PDB ID: 4NZ2) [50]. The inhibitor and water molecules were excluded. The CYP3A4 structure for the docking procedure was prepared from chain A of the complex of CYP3A4 with clotrimazole (PDB ID: 8SPD) [51]. The clotrimazole molecule and water molecules were removed. The docking was performed using the GOLD software (available within the Cambridge Structural Database software package, ver. 2026.1.0 [52]). The specific configurations of GOLD included the use of the chemscore_p450_csd template and parameters, definition of the cavity for docking included all the residues (a) constituting the hydrophobic cleft (V113, L201, N204, I205, L208, E300, and L366), the narrow solvent channel (L102 and R108), and the Phe-cluster (F100, F114 and F476) of the CYP2C9 [53], (b) main substrate channel 2e [54] of the CYP3A4 (R105, F108, G111, K115, I118, S119, I120, E122, R212, F213, F215, V240, and A370), the Chemscore scoring function was selected, and the GA setting was set to very flexible option (200% of the default search efficiency). The top-ranked solutions for both ligands (complexes **1** and **2**) were further graphically visualised by Discovery Studio Visualizer v19.1.0.18287 (©2018 Dassault Systèmes Biovia Corp., San Diego, CA, USA), and the interactions between the host protein and ligands were analysed and visualised by LigPlot+ v.2.3.1 [55].

## 3. Results

### 3.1. Pharmacokinetic Properties (ADME) of the Studied Complexes ***1*** and ***2***

**Figure 2:**
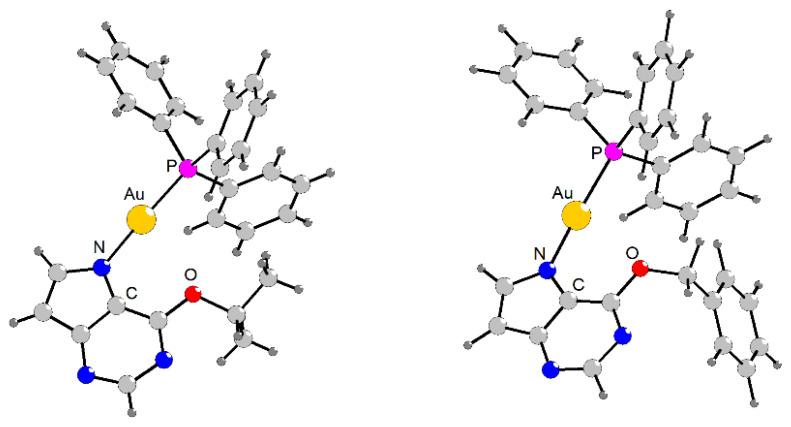
The molecular geometries of complexes **1** ((***left***), as determined using a single-crystal X-ray analysis as adopted from the literature [25]) and **2** ((***right***), as optimised using DFT calculations). The interatomic parameters around the gold(I) atoms are as follows: Au–N = 2.041 Å, Au–P = 2.227 Å, N–Au–P = 176.35° (for **1**), and Au–N = 2.037 Å, Au–P = 2.384 Å, N–Au–P = 178.08° (for **2**).

The four fundamental pharmacokinetic processes are absorption, distribution, metabolism, and excretion (ADME). The in vitro ADME properties of the investigated gold(I)–triphenylphosphine complexes **1** and **2** (see Figure 2) were assessed to obtain preliminary information on their pharmacokinetic behaviour and developability.

The assessed parameters included chemical stability in phosphate-buffered saline (PBS), plasma stability, microsomal stability, plasma protein binding (PPB), and passive permeability determined by PAMPA. The pharmacokinetic characteristics of the complexes are summarised in Table 1.

Both complexes demonstrated high chemical stability in PBS throughout the 120 min incubation period, with more than 90% of the parent compound remaining at all time points. Complex **1** retained 94.4 ± 3.3% and complex **2** retained 91.5 ± 1.2% of the initial concentration after 120 min. Plasma stability followed a comparable pattern: 90.9 ± 4.2% and 92.3 ± 2.2% of complexes **1** and **2**, respectively, remained intact after 120 min of incubation in human plasma. Incubation with human liver microsomes revealed a time-dependent decrease in the concentration of both complexes. After 60 min, 64.5 ± 2.3% of complex **1** and 50.6 ± 1.8% of complex **2** remained, corresponding to moderate intrinsic metabolic stability, according to Nassar [56]. Complex **2** exhibited a slightly faster rate of depletion compared to complex **1** throughout the entire incubation period. Both complexes exhibited high plasma protein binding, with bound fractions of 81.5 ± 3.1% for complex **1** and 79.5 ± 2.5% for complex **2**. No statistically significant difference in PPB was observed between the two compounds. The PAMPA assay yielded log Papp values of −7.8 ± 0.5 for complex **1** and −7.3 ± 0.1 for complex **2**. Based on the classification threshold established using the reference compounds propranolol (high permeability) and atenolol (low permeability), where compounds with log Papp > −5 are categorised as highly permeable [57], both complexes were classified as low permeability compounds.

**Table 1:** Pharmacological parameters of complexes **1** and **2**.

	% Compound Remaining							
	Chemical Stability				Plasma Stability			
Compound	15 min	30 min	60 min	120 min	15 min	30 min	60 min	120 min
complex **1**	99.2 ± 3.5	96.3 ± 3.3	95.4 ± 2.4	94.4 ± 3.3	99.9 ± 3.5	99.4 ± 1.5	95.7 ± 3.4	90.9 ± 4.2
complex **2**	99.8 ± 2.5	98.7 ± 0.5	92.2 ± 1.2	91.5 ± 1.2	96.1 ± 1.4	94.8 ± 3.3	94.7 ± 3.3	92.3 ± 2.2
	% Compound remaining							
	Microsomal stability			Plasma protein binding		PAMPA		
Compound	15 min	30 min	60 min	% Fraction bound		logP_app_	Category ^a^	
complex **1**	89.3 ± 3.1	81.4 ± 2.85	64.5 ± 2.3	81.5 ± 3.1		−7.8 ± 0.5	Low permeable	
complex **2**	85.5 ± 3.0	77.5 ± 1.71	50.6 ± 1.8	79.5 ± 2.5		−7.3 ± 0.1	Low permeable	

^a^ According to the logP_app_ obtained from the reference drugs, compounds with logPe > −5 were categorised as highly permeable, while those with logPe < −5 were considered as poorly permeable [57].

### 3.2. Effects of Complexes ***1*** and ***2*** and Their Free Ligands on Catalytic Activities of Cytochrome P450 in Human Liver Microsomes

**Figure 3:**
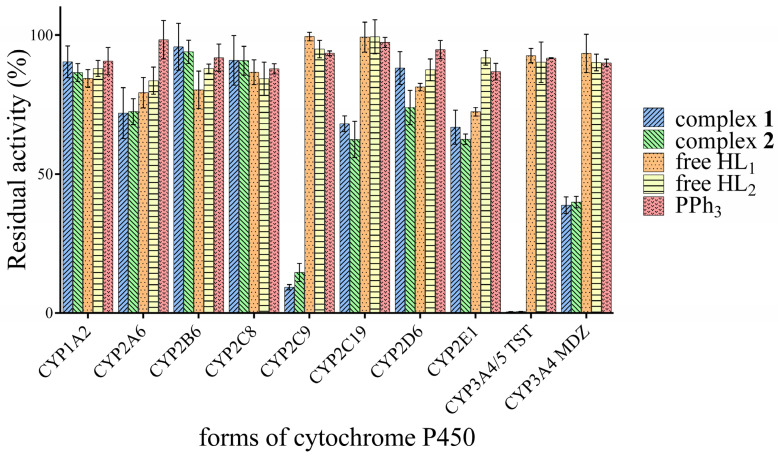
In vitro inhibitory effects of the studied compounds at 100 μM concentration on nine major human cytochrome P450 forms. Data are expressed as residual enzymatic activity (%) relative to control incubations without inhibitor. TST: testosterone, MDZ: midazolam, free HL_1_ = 6-isopropyloxy-9-deazapurine and free HL_2_ = 6-benzyloxy-9-deazapurine.

The inhibitory effects of complexes **1** and **2**, their corresponding free ligands HL_1_ = 6-isopropyloxy-9-deazapurine and HL_2_ = 6-benzyloxy-9-deazapurine, and triphenylphosphine (PPh_3_) were evaluated against nine major human cytochrome P450 isoforms. Specifically, the inhibition of activities of CYP1A2, CYP2A6, CYP2B6, CYP2C8, CYP2C9, CYP2C19, CYP2D6, CYP2E1, and CYP3A were evaluated. The tested compounds were studied at concentration levels of 0, 10, 25, 50, 75, and 100 μM. The activity of all tested CYP450 forms was significantly affected by the maximum concentration of the studied compounds (see Figure 3 for details). Results are expressed as a percentage of residual enzymatic activity.

Only weak to moderate inhibition was observed for CYP1A2, CYP2A6, CYP2B6, and CYP2C8. Residual activities for the gold complexes ranged predominantly between 70 and 95%, indicating limited interaction with these systems. The free ligands (HL_1_ and HL_2_) exhibited similar or slightly stronger inhibition than the complexes for some isoforms (e.g., CYP2B6), while PPh_3_ alone showed minimal inhibitory effect, particularly toward CYP2A6 (98% residual activity).

Moderate inhibition was observed for CYP2C19, with residual activities of approximately 62–68% for the complexes, while free ligands and PPh_3_ were essentially inactive (>97%). For CYP2E1, residual activity ranged from 62 to 67% for both complexes at 100 µM. Complex **2** exhibited stronger inhibition of CYP2D6 (74% residual activity) compared to complex **1** (decreased to 88% activity of the uninhibited enzyme).

A striking inhibitory effect on CYP2C9 was observed. Residual activity decreased to 9.3% for complex **1** (with IC_50_ 30.41 ± 1.74 μM) and 14.6% for complex **2** (IC_50_ 57.21 ± 2.90 μM). In contrast, both free HL_1_ and HL_2_ ligands and PPh_3_ maintained >93% enzymatic activity.

The most significant findings were obtained for CYP3A4/5. Enzyme activity was reduced to <1% in the testosterone 6β-hydroxylation assay and to around 40% in the midazolam 1′-hydroxylation assay for both complexes. The IC_50_ values for the testosterone 6β-hydroxylation assay were evaluated as 4.62 ± 1.31 μM and 3.92 ± 1.19 μM for complexes 1 and 2, respectively and 23.01 ± 2.23 μM for complex 1 and 33.70 ± 3.24 μM for complex 2 using the midazolam 1′-hydroxylation assay. In contrast, free ligands and PPh_3_ retained >89% activity in both assays.

**Figure 4:**
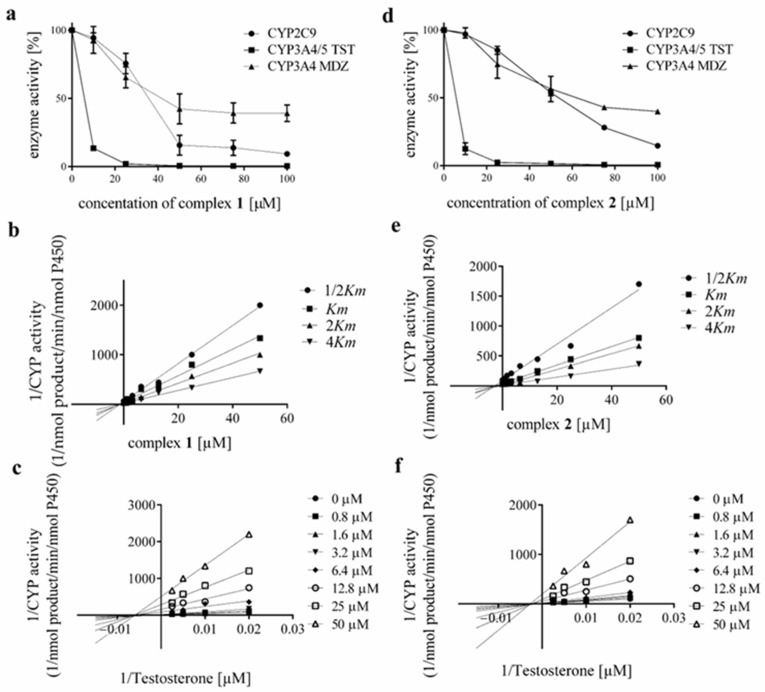
(**a**) The effect of complex **1** and (**d**) complex **2** on the enzymatic activity of CYP2C9 and CYP3A4/5 with the specific substrates of testosterone and midazolam in HLMs. Activity inhibition is determined as the mean of two independent experiments performed in triplicate ± SD and is expressed as a percentage of activity remaining relative to the control (100%, without the studied compound). Concentrations of complex **1** and **2** in the reaction mixture were 0, 10, 25, 50, 75 and 100 μM. (**b**,**e**) Dixon plot and (**c**,**f**) Lineweaver-Burk plot for inhibition of CYP3A4/5 (testosterone 6β-hydroxylation assay) enzymatic activity by complexes **1** and **2** at four substrate concentrations (50, 100, 200, 400 µM) for eight concentrations of complexes (0, 0.8, 1.6, 3.2, 6.4, 12.8, 25 and 50 μM).

Due to the significant inhibition observed for CYP2C9 and CYP3A4/5 (shown in more detail in Figure 4a,d for complexes **1**, and **2**, respectively), several experiments were performed to study the mechanism of inhibition and the influence of both complexes on the resulting *Ki* values. Dixon and Lineweaver-Burk plots were constructed using four substrate concentrations (corresponding to 1/2 *Km*, *Km*, 2 *Km*, and 4 *Km*) when the results for the CYP3A4/5 testosterone assay are shown in Figure 4b,c for complex **1**, and in Figure 4e,f for complex **2**. Complex **1** revealed a noncompetitive type of inhibition for two studied forms of cytochromes P450, with *Ki* values of 35.56 ± 2.69 μM for CYP2C9 and 5.12 ± 0.87 μM for CYP3A4/5 (testosterone 6β-hydroxylation assay). In the case of complex **2**, the course of these plots also indicates fully noncompetitive inhibition for CYP2C9 and CYP3A4/5, with *Ki* values of 56.39 ± 4.85 μM, and 4.51 ± 1.69 μM, respectively.

**Figure 5:**
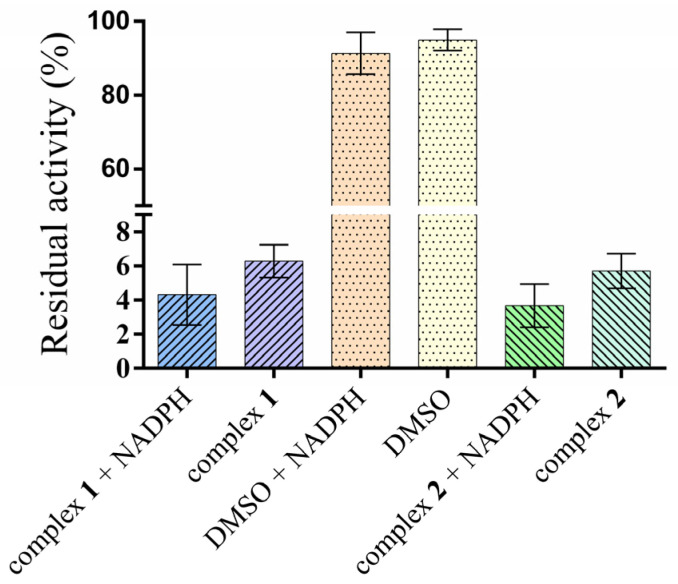
Single Point Assay of complexes **1** and **2**, and DMSO as a vehicle solvent, in the presence or absence of NADPH. In one case, the tested complexes were preincubated with NADPH and microsomes at a concentration 10 times higher than in conventional activity testing. In the second case, NADPH was added to the sample containing microsomes and the compounds only after preincubation.

Since metal-containing compounds have also been reported to cause time-dependent inhibition of CYP3A4 [58], a basic screening of potential inhibitors was performed to verify that the inhibitory potency of the compounds did not increase with increasing preincubation time, e.g., by the formation of an enzyme-binding product, and thus was not time-dependent. The studied complexes were subjected to a Single Point Assay to investigate a possible time-dependent mechanism in which the test compound is preincubated with microsomes in the presence or absence of NADPH. If there is a time-dependent inhibition, then a decrease in activity will be observed only in the presence of NADPH during preincubation. In parallel, possible inhibition by the solvent itself is also monitored. The data showed that neither the complexes nor the solvent used caused any time-dependent or irreversible inhibition (Figure 5).

### 3.3. Spectral Studies of the Interaction of the Tested Complexes ***1*** and ***2*** with Human Liver Microsomal CYP450

**Figure 6:**
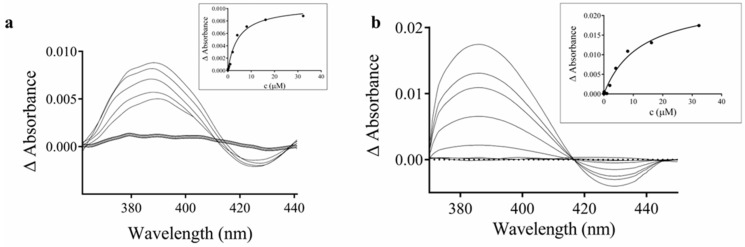
Difference spectra of the interaction of human liver microsomal cytochrome P450 with complex **1** (**a**) and complex **2** (**b**). CYP450 concentration was 1 μM, and concentrations of the tested complexes ranged from 0.002 to 33.31 μM in the cuvette. The insert presents a plot of the absorbance changes at 388 nm *versus* the concentration of the respective compounds.

The interaction of the studied Au(I) complexes with cytochrome P450 was further investigated with human liver microsomes using UV–Vis difference spectroscopy. This method enables monitoring ligand-induced changes in the heme environment of cytochrome P450 through alterations in the Soret absorption band, which is highly sensitive to modifications in the spin state and coordination of the heme iron. Both investigated complexes produced a characteristic type I difference spectrum, with a maximum at 388 nm and a minimum at 430 nm (Figure 6). Non-linear fitting of the absorbance changes as a function of ligand concentration enabled estimation of the spectral dissociation constants (*Ks*) [59]. The calculated values were 8.49 ± 1.67 µM for complex **1** and 12.62 ± 2.42 µM for complex **2**, indicating moderate binding affinity of both Au(I) complexes toward microsomal cytochrome P450.

### 3.4. Thermodynamic Characterization of Interaction Between Complexes and Cytochrome P450 Using Isothermal Titration Calorimetry (ITC)

The thermodynamic parameters governing the binding of complexes **1** and **2** to selected CYP450 forms were determined by isothermal titration calorimetry (ITC). The forms CYP1A2, CYP2A6, and CYP3A4 (both bactosomes and recombinant human protein) were selected for ITC analysis based on their differential susceptibility to inhibition by the studied complexes, as established in the preceding microsomal inhibition experiments. All thermodynamic parameters obtained by ITC determinations are summarised in Table 2.

**Figure 7:**
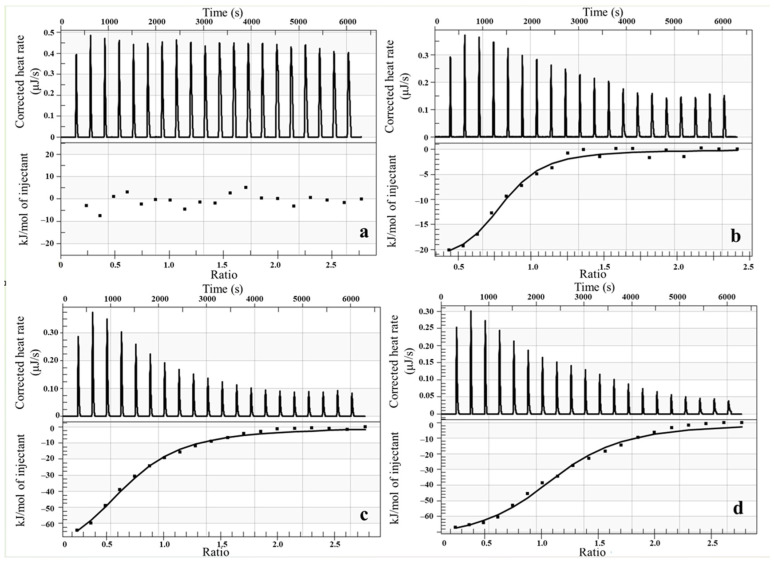
Representative ITC data for complex **1** binding to bactosome CYP1A2 (**a**), bactosome CYP2A6 (**b**), bactosome CYP3A4 (**c**), and recombinant human CYP3A4 (**d**). (**Top**) Raw data plot of heat flow against time for the titration of CYP450 with complex **1**. (**Bottom**) Plot of molar enthalpy change against complex **1**/cytochrome P450 molar ratio.

Titration of both complexes into bactosome CYP1A2 produced only a weak exothermic signal indistinguishable from the heat of dilution, and no significant binding interaction was detected for either compound (shown in Figure 7a for complex **1**.). Accordingly, no thermodynamic parameters could be extracted from these titrations. In contrast, titration into CYP2A6 bactosome yielded measurable exothermic signals, from which thermodynamic parameters were successfully determined (Figure 7b). Substantially stronger interactions were observed for CYP3A4. Representative calorimetric titration profiles for complex **1** with bactosomes and recombinant CYP3A4 are shown in Figure 7c,d; the solid lines represent the best fit of the experimental data to the independent binding sites model. As the binding sites on the enzyme became progressively occupied during the titration, the exothermicity of successive injection peaks decreased and eventually reached saturation, consistent with a well-defined binding process. The stoichiometry of binding was approximately 1:1 (complex: enzyme) for all interactions where binding was detected, with n values ranging from 0.942 to 1.437. All ΔG values were negative across all studied interactions.

### 3.5. Molecular Docking

**Figure 8:**
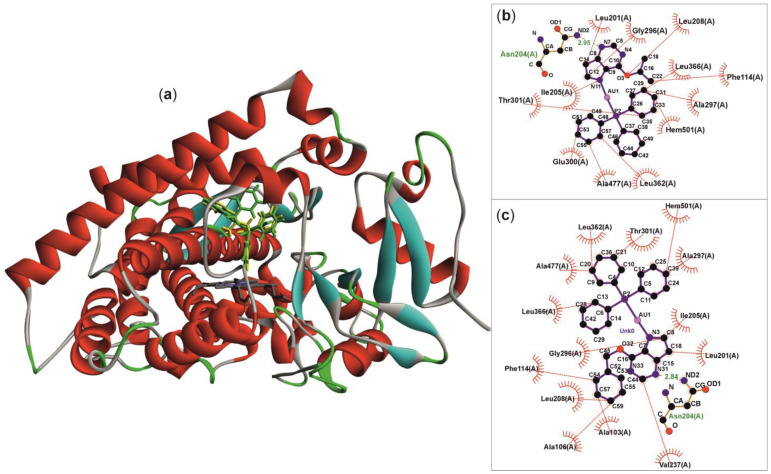
The comparison of the best-ranked docking results for complexes **1** (yellow-colored molecule) and complex **2** (green-colored molecule) within the active-site cavity of CYP2C9, shown in flat ribbon display style coloured by the secondary structure type (**a**), and LigPlot+ analysis of the interactions of **1** (**b**) and **2** (**c**), highlighting the hydrogen bonding between the N3-atom of the purine residue and Asn204 amino acid residue of the CYP450 protein and a series of hydrophobic interactions with the hydrophobic amino acid residues.

**Figure 9:**
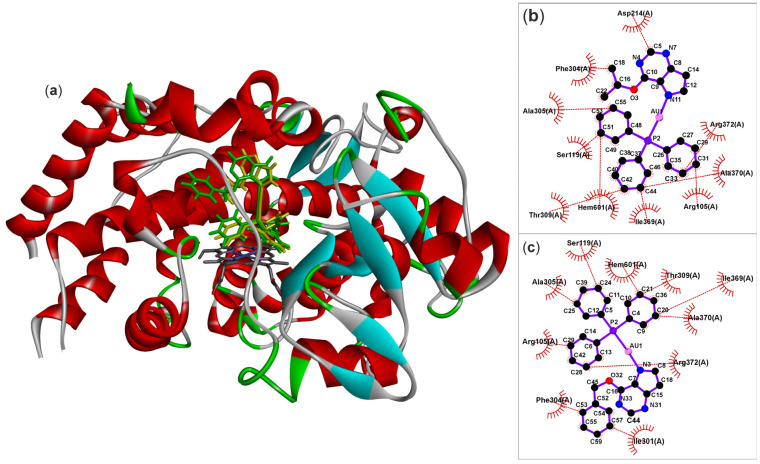
The comparison of the best-ranked docking results for complexes **1** (yellow-colored molecule) and complex **2** (green-colored molecule) within the active-site cavity of CYP3A4, shown in flat ribbon display style coloured by the secondary structure type (**a**), and LigPlot+ analysis of the interactions of **1** (**b**) and **2** (**c**), highlighting the major role of hydrophobic interactions with the hydrophobic amino acid residues.

The molecular docking confirmed that both gold(I) complexes studied can occupy the main substrate channels around the active sites of CYP2C9 (Figure 8a) and CYP3A4 (Figure 9a) and reasonably interact with their predominantly hydrophobic interiors (Figure 8b,c and Figure 9b,c), forming hydrophobic interactions with the hydrophobic side chains of several amino acid residues. In the case of CYP2C9, the position was stabilised by a hydrogen bond between the N3 nitrogen of the purine residue and the Asn204 residue, at a distance of 2.84–2.95 Å. In both cases, complex **2** showed higher affinity, with overall higher Chemscore values (49.42 for CYP2C9 and 46.85 for CYP3A4) than complex **1** (44.02 for CYP2C9 and 41.64 for CYP3A4). Taken together, the proposed mode of inhibition, based on limiting substrate access to the active site, may indeed be one of the mechanisms underlying the experimentally demonstrated non-competitive inhibition of both CYP2C9 and CYP3A4 by the studied complexes.

## 4. Discussion

In this study, two gold(I)-triphenylphosphine complexes containing 6-alkoxy-9-deazapurine ligands were investigated for their interaction with major drug-metabolising enzymes and their basic pharmacokinetic properties in vitro. A thorough understanding of pharmacokinetic behaviour is essential for evaluating the translational potential of novel metallodrug candidates, yet comprehensive in vitro ADME profiling of gold(I) complexes remains rare. The results obtained in this study provide a coherent pharmacokinetic picture for complexes **1** and **2** that can be rationalised in terms of the structural and electronic features of the Au(I)–PPh_3_ scaffold. The high chemical and plasma stability observed for both complexes (>90% over 120 min) is a favourable finding from a drug development perspective, as it indicates that neither rapid hydrolysis nor nonspecific degradation in the extracellular environment is likely to represent a limiting factor for systemic exposure. This behaviour is consistent with the well-established kinetic inertness of linear Au(I)–phosphine complexes, in which the thermodynamically strong Au–P bond and the neutral overall charge of the coordination sphere confer substantial resistance to ligand dissociation and aqueous decomposition [60]. From a practical standpoint, this stability profile suggests that the complexes would reach their intracellular targets in a largely intact form following systemic administration, a prerequisite for pharmacological activity that is not always met by more labile metal-based systems. The moderate microsomal stability observed for both complexes—with approximately 65% and 51% of the parent compound remaining after 60 min for complexes **1** and **2**, respectively—places them in the intermediate category according to established classification criteria [56]. This result warrants careful interpretation, as the metabolic pathways operative for gold-based complexes differ fundamentally from those governing classical organic drugs. While CYP450-mediated oxidative metabolism undoubtedly contributes to microsomal depletion, Au(I) complexes are additionally susceptible to non-enzymatic processes including thiol-mediated ligand exchange with glutathione and microsomal protein thiols, redox-driven transformations of the Au(I) centre, and oxidative modification of the phosphine ligand [61]. The slightly faster depletion of complex **2** relative to complex **1** may reflect the increased susceptibility of the benzyloxy group to oxidative *O*-debenzylation, a well-characterised CYP450-mediated metabolic pathway for benzylic ethers. High plasma protein binding (~80% for both complexes) is a characteristic shared by many lipophilic Au(I)–phosphine systems and can be attributed to two complementary mechanisms: hydrophobic partitioning of the PPh_3_ moiety into protein binding pockets, and the pronounced affinity of the Au(I) centre towards thiol-containing residues–particularly Cys-34 of human serum albumin [62]. This behavior is mechanistically analogous to that well-documented for Auranofin, the only clinically approved gold(I)–phosphine complex, which also displays extensive plasma protein binding driven by rapid Au(I) transfer to the Cys-34 thiol of human serum albumin [63]. Importantly, despite this high degree of protein binding, Auranofin retains clinical efficacy and achieves sufficient free drug concentrations to exert pharmacological effects in vivo, demonstrating that high PPB does not necessarily preclude clinical utility for gold(I) complexes [64]. The observed PPB values for complexes 1 and 2 are therefore fully comparable to those of the clinical reference compound and should not be regarded as an absolute pharmacokinetic barrier to further preclinical development.

Passive permeability, determined by PAMPA, categorized both complexes as low permeability compounds compared with the control compounds, propranolol and atenolol [57]. Although the triphenylphosphine fragment contributes to lipophilicity, the relatively large molecular size, steric bulk, and limited availability of polar functionalities may limit efficient passive diffusion across artificial membranes. Low PAMPA permeability has been reported for several metal-based complexes and does not necessarily preclude effective cellular uptake in biological systems [65]. Indeed, unlike classical small-molecule drugs that rely predominantly on transcellular passive diffusion, gold(I)–phosphine complexes have been shown to exploit active cellular uptake mechanisms, including copper transporter 1 (CTR1) and organic cation transporters (OCT1/OCT2), which have been implicated in the cellular accumulation of gold complexes and may substantially compensate for limited passive permeability [66]. Additionally, the considerable lipophilicity conferred by the triphenylphosphine moiety may facilitate membrane association and endocytotic uptake pathways that are not captured by the PAMPA model, which exclusively measures passive transcellular diffusion [67]. A directly analogous situation has been documented for Auranofin, which, despite suboptimal passive permeability, achieves effective intracellular concentrations through active transport mechanisms, yet retains potent biological activity both in vitro and in vivo [64].

The pharmacokinetic profile of complexes **1** and **2**, compared with Auranofin, is particularly interesting, as both display similarly extensive plasma protein binding and suboptimal passive permeability, yet achieve oral bioavailability of approximately 25% and clinically meaningful pharmacological effects. This precedent suggests that the pharmacokinetic limitations identified here do not necessarily preclude in vivo efficacy. Should further optimization be required, nanoparticle-based formulation strategies, including chitosan, PEG encapsulation and liposomal delivery systems, have been successfully employed to enhance bioavailability and tumor targeting of analogous gold(I) complexes while reducing systemic toxicity [68]. A consistent finding across all evaluated ADME parameters was the absence of pronounced differences between complexes **1** and **2**, despite the distinct steric and lipophilic character of their respective *O*-substituents (isopropyloxy versus benzyloxy). This observation strongly suggests that the pharmacokinetic behavior of these compounds is predominantly governed by the Au(I)–PPh_3_ core structure, with the peripheral heterocyclic substituent playing a secondary role. This conclusion has important implications for future medicinal chemistry optimisation, and the present in vitro data provide the essential pharmacokinetic and metabolic foundation for rational design of future in vivo studies.

The CYP450 inhibition profiling revealed a striking selectivity pattern that merits detailed discussion from both mechanistic and translational perspectives. The limited inhibition observed for CYP1A2, CYP2A6, CYP2B6, and CYP2C8 (residual activities 70–95% at 100 µM) indicates that neither the 9-deazapurine heterocyclic scaffold nor its coordination to the Au(I) centre produces significant interactions with these forms at pharmacologically relevant concentrations [69]. This finding is reassuring from a safety perspective, as it suggests a low risk of drug–drug interactions mediated through these enzymes. The observation that free ligands exhibited comparable or even marginally stronger inhibition than the intact complexes for some of these forms (e.g., CYP2B6), while PPh_3_ alone was essentially inactive, indicates that the weak interactions within this group are primarily attributable to the heterocyclic moiety rather than to the metal centre or the phosphine ligand. Comparable weak inhibition of these CYP450 isoforms has been reported for other Au(I)–phosphine complexes at similar concentrations [69]. The pronounced inhibition of CYP2C9, with residual activities as low as 9.3% for complex **1** and IC_50_ values in the range of 30–57 µM, represents a finding of considerable clinical significance that must be evaluated in the context of the therapeutic window of co-administered drugs most likely to be affected. CYP2C9 is responsible for the oxidative metabolism of numerous widely prescribed drugs, including warfarin, phenytoin, losartan, glipizide, and several non-steroidal anti-inflammatory drugs (NSAIDs), and is characterised by a relatively hydrophobic active-site cavity that preferentially accommodates lipophilic, anionic, or bulky substrates [32]. For several of these substrates, most notably warfarin, phenytoin, and glipizide, even moderate CYP2C9 inhibition can result in clinically significant elevations in plasma levels with associated toxicity risks including bleeding, seizures, or hypoglycaemia [70]. The fact that both free ligands and PPh_3_ individually maintained more than 93% enzymatic activity unambiguously demonstrates that the potent CYP2C9 inhibition is a property of the intact gold(I) complex and cannot be attributed to any single molecular component. This synergistic effect of metal coordination on CYP450 inhibition—where the assembled complex exhibits dramatically stronger inhibition than any of its constituent parts—represents a key finding of this study and highlights the importance of evaluating metallodrugs as complete entities rather than extrapolating from the properties of individual ligands. From a drug development perspective, the potent CYP2C9 inhibition observed here flags a potential risk for pharmacokinetic drug–drug interactions, particularly in clinical scenarios involving co-administration with narrow therapeutic index drugs such as warfarin. The most significant and mechanistically informative results were obtained for CYP3A4/5, the single most important drug-metabolising enzyme in humans, responsible for the biotransformation of approximately 30–50% of all clinically used drugs [71]. The near-complete suppression of testosterone 6β-hydroxylation (<1% residual activity; IC_50_ 4 µM) combined with considerably weaker inhibition of midazolam 1′-hydroxylation (40% residual activity; IC_50_~23–34 µM) reveals a pronounced substrate-dependent inhibition pattern. Substrate-dependent inhibition is a well-documented phenomenon for CYP3A4/5 and arises from the enzyme’s unusually large and flexible active-site cavity, which can accommodate multiple ligands simultaneously and supports several distinct binding orientations [72]. Testosterone and midazolam interact with partially overlapping but non-identical binding regions within the CYP3A4 active site. The dramatically stronger inhibition in the testosterone assay suggests that the Au(I)–PPh_3_ complexes interact preferentially with the hydrophobic binding region associated with steroid substrate recognition, likely driven by extensive hydrophobic contacts between the bulky, lipophilic PPh_3_ scaffold and the nonpolar residues lining this portion of the active cavity.

The noncompetitive mechanism of inhibition established through Dixon and Lineweaver–Burk analyses for both CYP2C9 and CYP3A4/5 provides crucial mechanistic insight. Noncompetitive inhibition implies that the inhibitor binds to the enzyme at a site distinct from the substrate-binding site, or to both the free enzyme and the enzyme–substrate complex with equal affinity, without directly competing for the catalytic centre. For CYP3A4/5, this is consistent with several structural possibilities: (*i*) binding at one of the known peripheral or allosteric sites that modulate catalytic activity without occupying the substrate-binding pocket; (*ii*) interaction with hydrophobic access channels that regulate substrate entry and product egress; or (*iii*) binding to surface regions that induce conformational changes propagating to the active site [73]. Given the considerable molecular dimensions and lipophilicity of the Au(I)–PPh_3_ scaffold, it is plausible that the complexes preferentially occupy peripheral hydrophobic regions rather than inserting into the immediate vicinity of the heme iron. As indicated by molecular docking results of complexes **1** and **2** into the main substrate channels of CYP2C9 and CYP3A4, this proposed mode of interaction, which limits substrate access to the active site, may indeed be one of the mechanisms underlying the experimentally demonstrated non-competitive inhibition of both CYP2C9 and CYP3A4. This interpretation is strongly supported by the UV–Vis spectroscopic data (discussed below), which indicate substrate-like binding within the hydrophobic cavity without direct coordination to the heme iron. The Ki values obtained for CYP3A4/5 (5.12 ± 0.87 µM for complex **1** and 4.51 ± 1.69 µM for complex **2** in the testosterone assay) fall within the low-micromolar range and are comparable to those reported for several clinically relevant CYP3A4 inhibitors. Importantly, noncompetitive inhibitors retain their inhibitory potency regardless of substrate concentration, which distinguishes them from competitive inhibitors whose effects can be overcome by elevated substrate levels. This characteristic may increase the clinical significance of the observed interaction, as inhibition would persist even under conditions of high substrate flux through CYP3A4.

A recurring and important theme throughout the CYP450 inhibition data is the clear demonstration that the intact Au(I) complex is the pharmacologically active species responsible for potent enzyme inhibition. For the most strongly affected forms, CYP2C9 and CYP3A4/5, free organic ligands and PPh_3_ individually showed negligible inhibition (>89–93% residual activity), while the assembled complexes produced IC_50_ values in the low-micromolar range. This observation has two important implications. First, it demonstrates that metal coordination fundamentally alters the interaction profile of the heterocyclic ligand with CYP450 enzymes, likely through changes in molecular geometry, electronic distribution, lipophilicity, and the introduction of novel binding determinants associated with the gold centre. Second, it validates the need to evaluate intact metal complexes in CYP450 inhibition assays, as predictions based on individual ligand properties would dramatically underestimate the interaction potential.

The confirmation that neither complex **1** nor complex **2** causes time-dependent (mechanism-based) inhibition of CYP3A4/5 is an important finding that favourably distinguishes these compounds from several known metallodrug systems. Time-dependent inhibition, which involves the formation of reactive metabolic intermediates capable of covalently binding to the enzyme, is associated with a disproportionately higher risk of clinically significant drug–drug interactions because the inhibitory effect persists beyond elimination of the parent compound and requires de novo enzyme synthesis for recovery [58]. The absence of this mechanism for complexes **1** and **2** indicates that the observed CYP3A4/5 inhibition is fully reversible, which is a more favourable safety profile from a drug development perspective. Furthermore, the high plasma protein binding (~80%) reduces the free inhibitor concentration available for CYP450 interaction [74], which, in combination with the reversible inhibition mechanism, places the DDI risk profile of these complexes in a significantly more manageable category compared to mechanism-based inactivators such as erythromycin or diltiazem [75]. From a pharmacodynamic perspective, the primary anticancer mechanism of complexes 1 and 2 operates through inhibition of thioredoxin reductase (TrxR), a selenocysteine-containing oxidoreductase overexpressed in many cancer cell types, leading to ROS accumulation, disruption of the thioredoxin redox cycle, and induction of apoptosis via mitochondrial pathways. The CYP450 interactions characterised here therefore represent secondary pharmacological interactions with implications primarily for drug–drug interaction risk rather than for the intended pharmacological effect, and in vivo pharmacokinetic studies will be essential to fully assess their clinical relevance.

UV–Vis difference spectroscopy provided complementary mechanistic insight into the nature of the interaction between the Au(I) complexes and microsomal cytochrome P450. Both complexes produced a characteristic type I difference spectrum, with an absorption maximum at 388 nm and a minimum at 430 nm, diagnostic of substrate-like binding that induces a low-spin-to-high-spin transition of the ferric heme iron by displacing the axially coordinated water molecule [59]. This spectral response provides direct evidence that the complexes interact with the enzyme by occupying the hydrophobic substrate-binding cavity rather than through direct metal–heme coordination, a finding fully consistent with the noncompetitive inhibition mechanism established in the kinetic analyses. Given the considerable steric bulk of the Au(I)–PPh_3_ scaffold, preferential occupancy of peripheral hydrophobic regions within the large and flexible CYP3A4/5 active site—rather than direct competition at the heme centre—represents the most plausible binding mode. The spectral dissociation constants (*Ks* = 8.49 ± 1.67 µM for complex **1** and 12.62 ± 2.42 µM for complex **2**) fall in the low-micromolar range, in good agreement with the IC_50_ and *Ki* values from the functional assays and provide independent confirmation of moderate binding affinity. The marginally lower *Ks* for complex **1** may reflect a slightly better steric fit of the more compact isopropyloxy substituent within the binding pocket compared to the bulkier benzyloxy group of complex **2**.

Taken together, the spectroscopic, kinetic, and time-dependency data converge on a coherent mechanistic model in which complexes **1** and **2** bind reversibly within the hydrophobic active-site cavity in a substrate-like, noncompetitive, and non-time-dependent manner, with the intact gold(I) complex being the pharmacologically relevant inhibitory species.

Finally, we utilised the ITC to analyse key thermodynamic parameters relevant to the binding of the complexes to cytochromes P450. The ITC data are consistent with the functional inhibition and spectroscopic results and provide mechanistic insight into the interaction of complexes **1** and **2** with CYP450 enzymes. The absence of detectable binding to CYP1A2 confirms that this form is not a relevant target, in agreement with the negligible inhibition observed in activity assays. For CYP2A6, moderate binding affinity (*Ka* ~ 10^6^ M^−1^) combined with positive entropy and modest enthalpy changes indicates a predominantly hydrophobic, surface-associated interaction, consistent with the weak inhibitory effect observed [76]. In contrast, significantly stronger binding was detected for CYP3A4 (*Ka* ~ 10^7^ M^−1^; *Kd* in the low nanomolar range), in excellent agreement with its pronounced inhibition. The larger enthalpic contribution and negative entropy changes (ΔS < 0) suggest a binding process accompanied by conformational restriction of the enzyme, consistent with the known structural flexibility of CYP3A4 and supporting a noncompetitive, allosteric mechanism [77]. The comparable thermodynamic profiles obtained for bactosomes and recombinant CYP3A4 confirm that the observed interactions are intrinsic to the enzyme. The approximately 1:1 stoichiometry and negative ΔG values further indicate a spontaneous and well-defined binding process. Overall, the ITC results support a coherent model in which the complexes bind to CYP3A4 preferentially through strong, reversible, noncompetitive binding. Overall, the combined pharmacokinetic, kinetic, thermodynamic, and spectroscopic data provide a consistent mechanistic picture of the interaction of these gold(I) complexes with CYP450 enzymes and highlight their relevance for further development as biologically active metal-based agents.

The present study addresses an important gap in the literature on gold(I)-based metallodrugs. While interactions of metal complexes with CYP450 enzymes have been reported for several other metal systems, including copper(II) phenanthroline complexes acting as irreversible competitive inhibitors of CYP1A1 [78], copper(II) quinolinonato complexes showing noncompetitive inhibition of CYP3A4/5 and CYP2C9 with type I binding spectra [44], platinum complexes interacting with multiple CYP450 isoforms [79], and iridium(III) and ruthenium(II) photosensor complexes binding within the CYP3A4 active site at nanomolar concentrations [40], but comparable data for gold(I)–phosphine complexes bearing 9-deazapurine-type ligands have not previously been reported. Crucially, unlike several of the copper(II) systems studied previously, where moderate complex stability raised the question of whether inhibition was attributable to the intact complex or to released metal ions [40,44], the high chemical and plasma stability of complexes **1** and **2** (>90% after 120 min) unambiguously identifies the intact Au(I) coordination compound as the pharmacologically active species. Furthermore, in contrast to most previous studies on gold(I) complexes that focused exclusively on cytotoxicity or target enzyme inhibition without metabolic characterization, the present work provides an integrated dataset combining CYP450 inhibition profiling across nine isoforms, inhibition mechanism characterization, direct enzyme binding analysis by both UV–Vis spectroscopy and ITC, and comprehensive in vitro ADME evaluation, representing the most complete pharmacokinetic characterization reported to date for Au(I)–phosphine complexes of this ligand class. The consistent finding that the Au(I)–PPh_3_ core, rather than the peripheral heterocyclic ligand, governs both the pharmacokinetic profile and the CYP450 interaction pattern has direct implications for the rational design of future gold-based metallodrugs, as it suggests that modifications of the 9-deazapurine scaffold can be exploited to optimize biological activity and selectivity without substantially altering the metabolic and safety profile of the compound class.

## 5. Conclusions

In this study, two gold(I)–triphenylphosphine complexes bearing 6-alkoxy-9-deazapurine ligands were comprehensively evaluated with respect to their in vitro pharmacokinetic properties and interactions with major drug-metabolising CYP450 enzymes. Both complexes demonstrated high chemical and plasma stability, moderate microsomal stability, substantial plasma protein binding, and low passive membrane permeability, a profile consistent with the physicochemical characteristics of the Au(I)–PPh_3_ scaffold. CYP450 inhibition profiling revealed selective and potent inhibition of CYP2C9 and CYP3A4/5, while the other tested forms were only weakly affected. Kinetic analyses established a noncompetitive mechanism of inhibition for both forms, and UV–Vis difference spectroscopy confirmed substrate-like, reversible binding within the hydrophobic active-site cavity without direct metal–heme coordination. ITC measurements provided thermodynamic evidence for strong, spontaneous, and enthalpically driven binding to CYP3A4, fully consistent with the functional and spectroscopic data. Notably, the intact gold(I) complex—rather than the individual ligand components—was identified as the pharmacologically active inhibitory species, highlighting the essential role of metal coordination in defining the biological interaction profile. The *O*-substituent on the 9-deazapurine backbone in the ligand had a minor influence on the overall pharmacokinetic and CYP450 inhibition profile, suggesting that the Au(I)–PPh_3_ core governs the dominant physicochemical and biological properties of this compound class. These findings contribute to a better understanding of the metabolic liabilities and potential drug interactions of gold(I) complexes and provide a valuable basis for their further rational development as metal-based bioactive agents.

## Figures and Tables

**Table 2:** Thermodynamic parameters defining the interactions of complexes **1** and **2** with CYPs based on ITC titrations.

		*Ka*	ΔH	n	*Kd*	ΔS	ΔG
		(1/M)	(kJ/mol)		(nM)	(J/mol.K)	(J/mol)
complex **1**	CYP1A2 ^B^	no interaction					
	CYP2A6 ^B^	5.08 × 10^6^	−21.01	0.974	196.8	77.06	−43,995.4
	CYP3A4 ^B^	9.05 × 10^6^	−53.22	1.125	110.5	−52.14	−47,376.9
	rCYP3A4	1.18 × 10^7^	−61.74	1.204	84.74	−71.72	−40,356.7
complex **2**	CYP1A2 ^B^	no interaction					
	CYP2A6 ^B^	3.25 × 10^6^	−19.62	1.256	307.7	58.26	−47,434.3
	CYP3A4 ^B^	2.35 × 10^7^	−62.59	0.942	42.5	−75.34	−47,300.7
	rCYP3A4	4.03 × 10^7^	−74.51	1.437	24.8	−158.9	−27,134.0

^B^ bactosomes; rCYP3A4 = recombinant human CYP3A4.

## Data Availability

The original contributions presented in this study are included in the article. Further inquiries can be directed to the corresponding author.

## Abbreviations

The following abbreviations are used in this manuscript:

ADME	Acronym of absorption, distribution, metabolism and excretion
Au(I)–NHC	Gold(I) *N*-heterocyclic carbene complex
CYP	Cytochrome P450
DMSO	Dimethyl sulfoxide
HLM	Human liver microsomes
HPLC	High-performance liquid chromatography
ITC	Isothermal titration calorimetry
MDZ	Midazolam
NADP^+^	Nicotinamide adenine dinucleotide phosphate
NADPH	Reduced nicotinamide adenine dinucleotide phosphate
NHC	*N*-heterocyclic carbene
PAMPA	Parallel artificial membrane permeability assay
PBS	Phosphate-buffered saline
PPB	Plasma protein binding
PPh_3_	Triphenylphosphine
RF-MS	RapidFire with tam
ROS	Reactive oxygen species
TrxR	Thioredoxin reductase
TST	Testosterone
